# Binocular visual function impairment is an independent risk factor in axial length growth and myopia progression

**DOI:** 10.3389/fmed.2025.1738844

**Published:** 2026-01-05

**Authors:** Shuyang Guo, Jingrong Liu, Hairong Cai, Ziyi Li, Yong Fang, Xingtao Zhou, Yang Shen, Wen Wen

**Affiliations:** 1 Department of Ophthalmology & Visual Science, Eye & ENT Hospital, Shanghai Medical College, Fudan University, Shanghai, China; 2 State Key Laboratory of Medical Neurobiology and MOE Frontiers Center for Brain Science, Institutes of Brain Science, Fudan University, Shanghai, China; 3 Key Laboratory of Myopia, Ministry of Health, Fudan University, Shanghai, China; 4 Shanghai Key Laboratory of Visual Impairment and Restoration, Fudan University, Shanghai, China; 5 Shanghai Aijiatongxin Ophthalmology Clinic, Shanghai, China; 6 Shanghai EVIS Technology Co., Ltd., Shanghai, China

**Keywords:** axial length, binocular visual function, myopia, risk factor, screening

## Abstract

**Purpose:**

In order to examine the association between binocular visual function (BVF) impairment and myopia progression, as well as its relationship with axial length (AL), spherical equivalent (SE), and axial length to-corneal radius (AL/CR) ratio.

**Methods:**

This 9-month longitudinal study involved 949 children [505 males; mean age: 10.96 ± 0.53 years; baseline SE: −0.62 (−1.38, 0.00); baseline AL: 23.80 ± 0.84]. Participants were classified based on the results of three BVF screenings (suppression, simultaneous vision, fusion vision, and stereopsis): those with normal BVF in all screenings were placed in the normal group, those with abnormal BVF in all screenings in the dysfunction group, and the rest in the unstable group. Linear mixed-effects models were used to assess the relationship between BVF status and changes in myopia indicators.

**Results:**

In the fully adjusted model, BVF status was significantly associated with rapid progression of AL, demonstrating a clear dose-response relationship. The annualized AL growth rate was 0.184 mm/year in the normal group, 0.226 mm/year in the unstable group, and 0.521 mm/year in the dysfunction group. All pairwise comparisons of the growth rates were statistically significant (normal vs. unstable: β = −0.00346 mm/month; normal vs. dysfunction: β = −0.02810 mm/month; unstable vs. dysfunction: β = −0.02463 mm/month; all *p* < 0.001). In contrast, BVF status showed no significant impact on the progression of SE or AL/CR ratio.

**Conclusion:**

BVF impairment is an independent risk factor for rapid AL growth, with a clear dose-response relationship. BVF screening could be integrated into routine eye examinations for school-aged children, allowing for early identification of individuals at high risk for rapid myopia progression. Incorporating BVF management into current myopia control strategies could lead to more effective, personalized interventions.

## Introduction

Myopia represents a significant global public health challenge (1). This trend is particularly pronounced in East Asia, where the prevalence of myopia among students in China increases rapidly with age. According to the latest data released by the National Health Commission of the People's Republic of China, the prevalence rates of myopia among elementary, middle, and high school students are 35.6, 71.1, and 80.5%, respectively. Given the irreversible retinal changes and substantial societal burden associated with high myopia, it is critical to identify modifiable risk factors and establish early intervention strategies during the key period of myopia progression—elementary school years.

The commonly used clinical interventions for myopia, including corrective lenses, atropine medication, and increased outdoor activities, each face specific limitations. For instance, corrective lenses offer only limited delay in progression (2), atropine use is limited by dose-dependent side effects and rebound after discontinuation (3), and increased outdoor time provides minimal protection for children who are already myopic (4). It is estimated that by 2050, unless new strategies to combat myopia are formulated, half of the global population will suffer from myopia (5). This critical situation necessitates the scientific community to identify more comprehensive risk factors for myopia progression and to develop more effective intervention methods.

Currently, the etiology of myopia remains unclear. The prevailing view is that the main risk factors are prolonged near work (6) and insufficient outdoor activity (7). However, other studies indicate that even reducing near work and increasing outdoor time may not effectively slow myopia progression (8). Research employing multiple regression models has demonstrated that leisure reading, other near activities, and outdoor activities showed no significant association with annual myopia progression (9). These findings indicate that unknown risk factors may contribute to myopia progression.

Generally, strabismus are disorders of neurovisual development resulting from abnormal visual experiences. Previous study (10) show that the prevalence of myopia (12.7%) in children with exotropia is significantly higher than in children without exotropia (4.6%). Due to the misalignment of the eyes, strabismus is often accompanied by binocular visual function (BVF) impairment (11). These findings suggest that BVF impairment may be associated with progression of myopia.

Additionally, research has already revealed a link between axial length (AL) growth and a decline in stereopsis acuity (12). A randomized controlled trial (RCT) shows that improving accommodative function via naked-eye 3-Dimensional (3D) training can significantly slow the AL elongation in myopic patients (13). These findings suggest that myopia and BVF impairment might share some parallel pathophysiological mechanisms. However, no large-scale prospective studies exist on BVF and myopia, and no study has examined how different BVF statuses differentially affect myopia progression. Moreover, it remains unclear whether BVF statuses affects various myopia progression indicators differently, especially AL, which reflects ocular biological growth, and spherical equivalent (SE), which reflects optical changes. Understanding these relationships is essential for clarifying the mechanisms by which BVF impairment contributes to myopia progression.

This cohort, longitudinal study aims to address three key questions: first, to determine if BVF impairment is an independent risk factor for myopia; second, to investigate whether varying levels of BVF impairment exert differential effects on myopia progression; and third, to identify which myopia-related biological parameter is most closely associated with BVF.

## Materials and methods

### Study design

**Figure 1:**
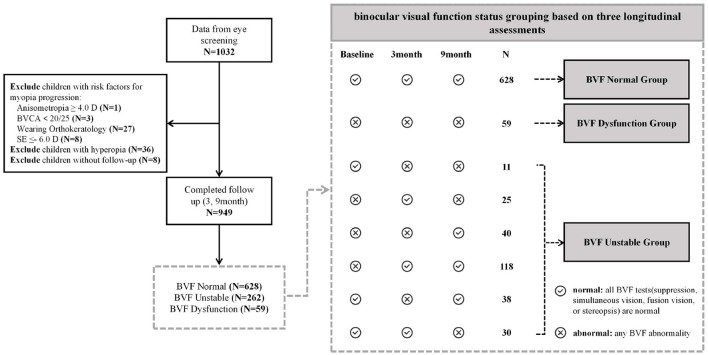
Study flowchart of participant enrollment and BVF grouping.

This school-based longitudinal observational study initially recruited 1,032 children in Grades 3 and 4 from a primary school in Shanghai, China, during a cross-sectional survey in February 2024. All participants were followed for 9 months, with data collected at baseline, 3, and 9 months. The exclusion criteria included: hyperopia, use of orthokeratology lenses, amblyopia, anisometropia >4.0 D, SE ≤ −6.0 D, and children who were lost to follow-up due to transfer, schedule conflicts, or absence. Ultimately, 949 participants were included in this analysis, resulting in a total of 2,847 valid observations (see Figure 1). The study was approved by the Ethics Committee of the Eye, Ear, Nose and Throat Hospital of Fudan University (No. 2023069) and was conducted in accordance with the Helsinki Declaration.

### Examinations

All examinations were performed by 15 trained specialists. At baseline and each follow-up visit, all children underwent a comprehensive ophthalmic examination, including: visual acuity, non-cycloplegic autorefraction, BVF assessment, ocular alignment assessment, and optical biometric measurements.

Visual acuity was measured at 3 m using the standard logarithmic E-chart. Non-cycloplegic autorefraction was conducted using an autorefractor (FAL-5000P, China), and optical biometry was performed using an optical biometer (FAL-1000A, China). The final analysis included data on SE, AL, and corneal curvature radius (CR). The axial length to-corneal radius (AL/CR) ratio was calculated as the ratio of AL to CR. Refractive error was calculated as sphere plus half negative cylinder. Ocular alignment was assessed using the alternate cover test and the Hirschberg test.

The BVF status was evaluated using a naked-eye 3D BVF testing device (EVIS Co., Inc., Shanghai, China). This device integrates naked-eye 3D technology with eye-tracking mechanisms, and the test content is presented as animations and games on a 15.6-inch naked-eye display. The subjects sat 50 cm away from the screen, keeping their heads still and stably focusing on the screen. The device sequentially assessed the subjects' suppression, simultaneous vision, fusion vision, and stereopsis. During the entire examination, the monitoring system ensured that the participants could stably position their chin and forehead on the device, keeping their heads still, and also monitored whether they were following the instructions to focus on the screen. If a participant did not meet the requirements, the test would automatically stop. Trained specialists would then communicate verbally with the participant to help them understand the procedure and encourage their cooperation. After this communication, all participants successfully completed the experiment.

### Definition

Hyperopia was defined as SE ≥ +1.00 D, emmetropia as SE > −0.50 and < +1.00 D, and myopia as SE ≤ −0.50 D. Astigmatism was defined as a cylinder power ≥ ±1.00 D. Anisometropia was determined by the interocular difference in SE, with a difference ≥ 1.00 D. Strabismus included both latent and manifest forms. Manifest strabismus was defined as any ocular deviation at 33 cm, with or without correction, and classified as esotropia or exotropia according to the direction of the primary deviation. Latent strabismus was identified using the alternate cover test and categorized as esophoria or exophoria based on the direction of the covered eye movement.

The categorization of grade levels was based on two groups: lower grades, which included Grade 3 students, and Upper grades, which included Grade 4 students.

Participants were classified into three distinct groups based on their BVF outcomes across all three screenings. In each BVF screening, if any BVF abnormality (suppression, simultaneous vision, fusion vision, or stereopsis) is present, the test is considered “abnormal”; if all tests are normal, the test is considered “normal.” The normal group included participants with consistently “normal” BVF results at each visit, the dysfunction group comprised those with consistently “abnormal” BVF results, and the unstable group consisted of participants who exhibited a mix of “normal” and “abnormal” BVF results over the three visits (Figure 1).

### Statistical analysis

Data analysis was ultimately based on the optical biometric parameters of the right eye. To ensure the methodological validity of using right-eye data alone, we evaluated potential interocular differences. Paired *t*-tests were conducted to compare baseline AL and SE between the right and left eyes. The results showed no significant differences at baseline for AL (mean difference = 0.0032 mm, *p* = 0.7255) or SE (mean difference = 0.0238 D, *p* = 0.3299). In addition, linear mixed-effects models with eye-side × time interaction terms were used to examine longitudinal changes. No significant differences in progression rates were found between eyes for AL (interaction coefficient = 0.0003 mm/month, *p* = 0.7755) or SE (interaction coefficient = 0.0063 D/month, *p* = 0.0886). These results support the assumption that the developmental trajectories of both eyes are parallel during myopia progression.

All statistical analyses were performed using R software (version 4.5.1). Normally distributed variables were expressed as mean ± standard deviation, while non-normally distributed variables were expressed as median (interquartile range); categorical variables were described as frequency (percentage).

To analyze the effect of BVF status on the longitudinal changes in AL, SE, and AL/CR ratio, we constructed a linear mixed-effects model, with random intercepts at the subject level. Before constructing the fully adjusted model, multicollinearity was assessed using the variance inflation factor (VIF), and variables with a VIF < 5 were included in the model. A stepwise adjustment strategy was applied.

The AL growth rate, defined as the monthly change in axial length, was derived from the model coefficients. Specifically, the coefficient for the continuous time variable represented the monthly growth rate for the Normal BVF group (reference). The interaction terms (BVF status × time) quantified the difference in growth rate between each group and the reference. Thus, the absolute growth rate for any group was obtained by adding its respective interaction term coefficient to the reference group's time coefficient.

Model 1 included BVF status, time (as a continuous variable), and their interaction (BVF status × time). Model 2 added age, gender, grade, and their interaction with time to Model 1. Model 3 further adjusted for refractive correction status, astigmatism, anisometropia, and strabismus, and their interactions with time.

Model fitting was performed using maximum likelihood estimation. Likelihood ratio tests were used to evaluate the goodness-of-fit for nested models. When significant BVF status × time interactions were found, marginal means for each BVF group were calculated using the emmeans package, followed by pairwise *post-hoc* comparisons. All *p*-values for comparisons were adjusted using the Benjamini-Hochberg method to control the false discovery rate. Statistical significance was set at a two-sided α = 0.05.

## Results

A total of 949 school-aged children (505 males; mean age: 10.96 ± 0.53 years) were included in this study, completing 2,847 follow-up observations over the 9-month period (Figure 1). Based on their baseline BVF status, participants were categorized into three groups: normal group (*n* = 628), unstable group (*n* = 262), and dysfunctional group (*n* = 59). Regarding refractive status, 512 participants were myopic, including 462 with mild myopia (SE ≤ −0.5 and > −3.0 D) and 50 with moderate myopia (SE ≤ −3.0 and > −6.0 D). The remaining 437 participants were emmetropic, of whom 430 were in the pre-myopia stage (SE ≤ +0.75 and > −0.5 D).

As shown in Table 1, no significant differences were observed among the three BVF groups in age, gender, or grade distribution (all *p* > 0.05). However, the dysfunction group exhibited significantly longer baseline AL (23.93 ± 0.68 vs. 23.75 ± 0.81 mm, *p* = 0.051), more negative SE (median: −0.875 vs. −0.500 D, *p* = 0.001), and higher AL/CR ratio (3.06 ± 0.10 vs. 3.03 ± 0.09, *p* = 0.005). This group also had higher proportions of anisometropia (30.5 vs. 11.8%, *p* < 0.001) and uncorrected myopia (39.0 vs. 22.6%, *p* = 0.002).

**Table 1:** Characteristic of school student at baseline.

**Characteristic**	**Normal**	**Unstable**	**Dysfunction**	***p*-value**
	***N*** = **628**	***N*** = **262**	***N*** = **59**	
**Gender, no. (%)**				
Male	347.0 (55.3%)	124.0 (47.3%)	34.0 (57.6%)	0.076^*^
Female	281.0 (44.7%)	138.0 (52.7%)	25.0 (42.4%)	
Age, mean ± SD, years	10.96 (0.53)	10.93 (0.51)	10.98 (0.56)	0.727^†^
**Grade, no. (%)**				
Lower grades	293.0 (46.7%)	117.0 (44.7%)	28.0 (47.5%)	0.843^*^
Upper grades	335.0 (53.3%)	145.0 (55.3%)	31.0 (52.5%)	
**Spectacle, no. (%)**				
Emmetropia	331.0 (52.7%)	108.0 (41.2%)	23.0 (39.0%)	0.002^*^
Myopia with spectacle	155.0 (24.7%)	70.0 (26.7%)	13.0 (22.0%)	
Myopia without spectacle	142.0 (22.6%)	84.0 (32.1%)	23.0 (39.0%)	
Anisometropia, no. (%)	74.0 (11.8%)	51.0 (19.5%)	18.0 (30.5%)	< 0.001^*^
Astigmatism, no. (%)	104.0 (16.6%)	49.0 (18.7%)	14.0 (23.7%)	0.33^*^
Strabismus, no. (%)	119.0 (18.9%)	51.0 (19.5%)	14.0 (23.7%)	0.674^*^
Baseline AL, mean ± SD, mm	23.75 (0.81)	23.88 (0.94)	23.93 (0.68)	0.051^†^
Baseline SE, median (P25, P75), dioptor	−0.50 (−1.25, 0.00)	−0.68 (−1.67, 0.13)	−0.88 (−1.56, 0.03)	0.001^‡^
Baseline AL/CR ratio, mean ± SD, ratio	3.03 (0.09)	3.04 (0.11)	3.06 (0.10)	0.005^†^

Abbreviations: BVF, binocular visual function; AL, axial length; SE, spherical equivalent; AL/CR ratio, axial length to-corneal radius ratio; SD, standard deviation.

^*^Pearson's Chi-squared test.

^†^One-way analysis of means.

^‡^Kruskal-Wallis rank sum test.

### Impact of BVF status on AL growth

**Figure 2:**
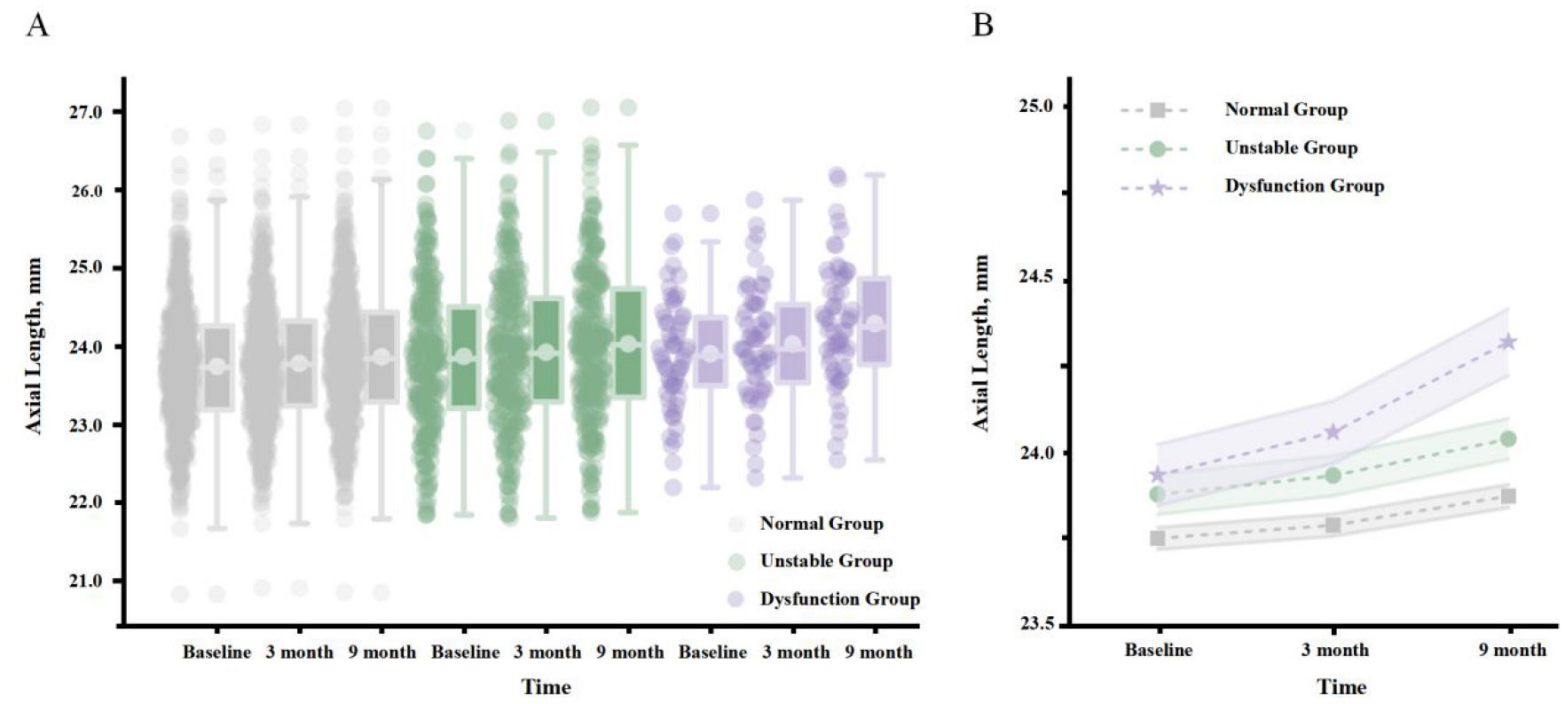
Longitudinal changes in AL by BVF groups. **(A)** Scatter points and box plots showing the distribution of axial length at each time point. **(B)** Line graph showing the mean AL with standard error at each time point.

Linear mixed-effects model analysis revealed a significant interaction between BVF status and time (*p* < 0.001) after adjusting for a range of confounding factors, including age, gender, grade, refractive correction status, astigmatism, anisometropia, and strabismus. This indicates a significant difference in the rate of AL growth among children with different BVF status (Figure 2, Table 2).

**Table 2:** Linear mixed model results for BVF effects on AL.

**Variable**	**Model 1 Coefficients (spherical error)**	**Model 2 Coefficients (spherical error)**	**Model 3 Coefficients (spherical error)**
BVF unstable (vs. normal)	0.1286 (0.0624)^*^	0.1769 (0.0589)^**^	0.1139 (0.0538)^*^
BVF dysfunction (vs. normal)	0.1825 (0.1156)	0.1670 (0.1088)	0.1097 (0.0997)
Time trend (per month)	0.0137 (0.0004)^***^	0.0314 (0.0144)^*^	0.0263 (0.0143)
BVF unstable × time	0.0042 (0.0008)^***^	0.0041 (0.0008)^***^	0.0035 (0.0008)^***^
BVF dysfunction × time	0.0292 (0.0015)^***^	0.0292 (0.0015)^***^	0.0281 (0.0015)^***^
AIC	−1,236.8	−1,344.8	−1,555.1
BIC	−1,189.2	−1,261.5	−1,412.2
Log likelihood	626.4	686.4	801.6

Model 1: basic model; Model 2: adjusted for age, gender, grade; Model 3: fully adjusted for age, gender, grade, spectacle use, astigmatism, anisometropia, and strabismus.

BVF, binocular visual function; AL, axial length; AIC, akaike information criterion; BIC, bayesian information criterion.

^*^p < 0.05.

^**^p < 0.01.

^***^p < 0.001.

In the fully adjusted model (Model 3), the interaction coefficients of unstable and dysfunction groups with time were 0.0035 mm/month (*p* < 0.001) and 0.0281 mm/month (*p* < 0.001), respectively. *Post-hoc* analysis (Table 3) further confirmed that all pairwise comparisons between groups were statistically significant (all *p*-values after FDR correction < 0.001), with a clear dose-response relationship: the dysfunction group showed the fastest growth, followed by the unstable group, and the normal group exhibited the slowest growth.

**Table 3:** Pairwise comparisons of AL growth rates among different BVF groups.

**Group comparison**	**Model 1 Coefficients (spherical error)**	**Model 2 Coefficients (spherical error)**	**Model 3 Coefficients (spherical error)**
Normal vs. unstable	−0.00418 (0.00080)^***^	−0.00409 (0.00080)^***^	−0.00346 (0.00080)^***^
Normal vs. dysfunction	−0.02916 (0.00148)^***^	−0.02920 (0.00148)^***^	−0.02810 (0.00148)^***^
Unstable vs. dysfunction	−0.02499 (0.00157)^***^	−0.02510 (0.00157)^***^	−0.02463 (0.00156)^***^

All comparisons FDR-adjusted.

BVF, binocular visual function; AL, axial length.

^***^p < 0.001.

From a clinical perspective (Table 4), the difference in growth rates was highly significant. Based on the marginal estimates from the fully adjusted model, the annualized AL growth rates for the BVF normal, unstable, and dysfunction groups were 0.184, 0.226, and 0.521 mm/year, respectively. The AL growth in the dysfunction group was 2.84 times that of the normal group.

**Table 4:** Clinical significance of AL growth rates by different BVF status.

**BVF status**	**Monthly growth rate (mm/month)**	**Annual growth rate (mm/year)**	**Fold change vs. normal**
Normal	0.0153	0.184	1 [reference]
Unstable	0.0188	0.226	1.23
Dysfunction	0.0434	0.521	2.84

Based on marginal means from fully adjusted model (Model 3).

BVF, binocular visual function; AL, axial length.

**Figure 3:**
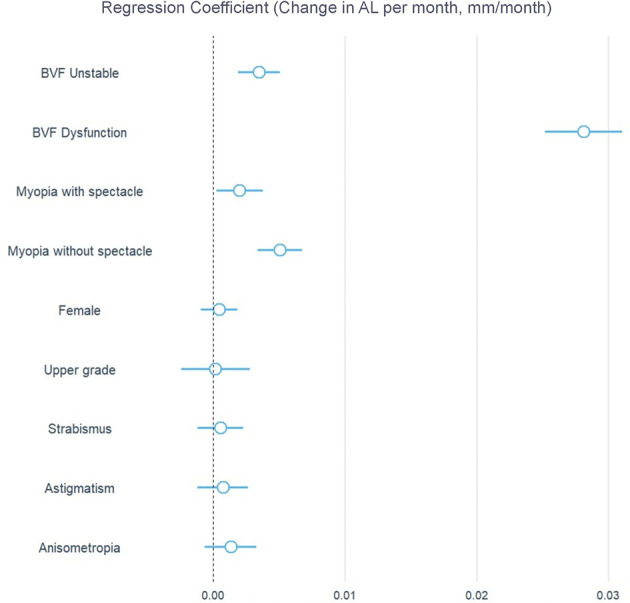
Forest plot of factors associated with AL growth rate. Associations are presented as regression coefficients (with 95% confidence intervals) for the interaction terms between each variable and time from the fully adjusted linear mixed model (Model 3). The reference groups are: BVF normal, emmetropia, male, lower grades, no strabismus, no astigmatism, and no anisometropia.

The forest plot visually demonstrates the relative effect sizes of various covariates on AL growth (Figure 3). This clearly shows that, after adjusting for other covariates, BVF dysfunction is one of the most significant risk factors in the interaction with time.

### Impact of BVF status on SE and AL/CR ratio

In contrast to the AL findings, BVF status had no significant effect on the rate of progression of SE and AL/CR ratio. In all adjusted models, the interaction between BVF status and time was not significant (all *p* > 0.05), and *post-hoc* comparisons showed no statistically significant differences between groups (see Supplementary Tables 1–6, Supplementary Figures 1–4).

## Discussion

This longitudinal study provides the first systematic evidence demonstrating that overall BVF status is an independent and significant risk factor for rapid AL elongation in school-aged children. We observed a pronounced dose-response relationship that the severity of BVF impairment was positively correlated with the rate of AL growth. Notably, this association was specific to biological AL, as no significant effect was found between BVF and other myopia-related biological parameters, including SE and AL/CR ratio. These findings provide compelling evidence supporting BVF as a potential marker for myopia progression and underscore its independent role in AL growth and myopia progression.

In the context of childhood myopia assessment, AL, SE, and AL/CR ratio are considered core biometric parameters (14). Among these, AL is arguably the most direct and robust indicator of myopia progression (15). AL's measurement can be rapidly and accurately performed through non-contact optical biometry without cycloplegia, demonstrating significant advantages over SE in large-scale screening and long-term monitoring in public health settings (16). Our finding that BVF abnormalities selectively influence AL growth, without a corresponding immediate change in SE, underscores its primary impact on the structural development of the eye. This dissociation suggests that the initial impact of BVF abnormalities may be on axial elongation, with optical components (such as the cornea and lens) potentially compensating in the short term, a nuance that SE alone may fail to capture. This highlights the superior sensitivity of AL for monitoring early, active growth phases in both public health screenings and long-term monitoring efforts.

Linear mixed-effects model analysis revealed a significant interaction between BVF status and time (*p* < 0.001) after adjusting for a range of confounding factors, including age, gender, grade, refractive correction status, astigmatism, anisometropia, and strabismus. This indicates a significant difference in the rate of AL growth among children with different BVF status. Furthermore, it is noteworthy that while baseline AL differed among groups in unadjusted analyses (Table 1), the main effects of BVF group (i.e., the baseline differences) became non-significant in the fully adjusted model (Model 3, Table 2). This statistical refinement underscores that the predominant influence of BVF dysfunction lies in its association with an accelerated rate of axial elongation over time, rather than in explaining pre-existing differences in axial length at the study's outset. This observation powerfully reinforces our central conclusion that BVF status is a critical determinant of growth rate.

During data collection, a total of 249 participants were myopic but without spectacles. To balance the accuracy and efficiency of screening, these participants underwent BVF testing under uncorrected refractive conditions. It is therefore possible that some BVF abnormalities originated from uncorrected refractive error. To control for this potential confounder, we included refractive correction status and its interaction with time (refractive correction status × time) as covariates in the Model 3. The final model confirmed that the effect of BVF abnormalities on AL elongation (dysfunction × time: β = 0.0281, *p* < 0.001; unstable × time: β = 0.0035, *p* < 0.001) was independent of refractive correction status. Although uncorrected refractive error itself also had a significant effect (β = 0.0051, *p* < 0.001), its magnitude was much smaller. These results indicate that BVF abnormalities is a stronger and independent risk factor for AL elongation beyond the influence of refractive correction.

Among the 249 myopic participants who were uncorrected at baseline, 64 (approximately 25.7%) commenced optical correction at subsequent visits, while 185 (74.3%) remained uncorrected. Our statistical model, which utilized baseline refractive correction status and its interaction with time as a covariate, did not fully capture the impact of this status change for a subset of participants. It could be postulated that this might influence the interpretation of the association between BVF abnormalities and AL elongation. However, we reason that this likely leads to a conservative bias in our estimates. The initiation of optical correction is expected to slow AL elongation. Since participants who began wearing spectacles were still analyzed within the “uncorrected” group, their potentially reduced growth rate would dilute the overall observed association in that group. Consequently, the effect sizes reported for both uncorrected refractive error (β = 0.0051) and, more importantly, binocular visual dysfunction (β = 0.0281) may underestimate the true strength of these associations. The fact that the effect of binocular visual dysfunction remained substantially larger (5.5 times) than that of uncorrected refractive error itself, even under these potentially diluting conditions, strengthens the conclusion that BVF abnormalities is an independent and potent risk factor for AL growth.

In this screened population, we observed that a substantial proportion of participants (321/949) were classified as having BVF abnormalities. Furthermore, an analysis of baseline distributions revealed that the proportion of normal BVF was higher in the strabismus group (64.7%) than in the anisometropia group (51.7%). This discrepancy can be explained by our operational definitions. The definition of strabismus in this large-scale screening context was broad, encompassing any detectable eye movement during the alternate cover test, which likely included individuals with small-angle, well-compensated deviations that may not severely disrupt BVF.

The conclusions of this study are strongly aligned with previous observational and interventional research on BVF and myopia. One study (12) reported a correlation between AL growth and the decline in stereopsis. Our study refines this association by exploring overall BVF status, quantifying its risk gradient, and revealing a dose-response relationship between BVF abnormalities and AL growth. Individuals with BVF impairment, include unstable and dysfunction, exhibited faster AL growth rates than those with unstable or normal BVF. Xie et al. (13), through a randomized controlled trial, confirmed that naked-eye 3D training can significantly delay AL growth.

A study (17) combining electroencephalography (EEG) and functional near-infrared spectroscopy (fNIRS) explored the neurophysiological mechanisms through which 3D visual training delays myopia progression. The researchers conducted naked-eye 3D training with 38 myopic participants, recording brain activity before and after the training. The results revealed a negative correlation between the improvement in static brain network efficiency and accommodative amplitude, while a positive correlation was observed between the enhancement of dynamic neurovascular coupling and the improvement in accommodative amplitude. This study demonstrates that 3D visual training improves binocular accommodation in myopic individuals by enhancing brain network efficiency and neurovascular coupling. As accommodative lag may contribute to axial elongation and myopia progression (18). Based on our understanding of the literature and our research findings, we propose a mechanistic hypothesis linking BVF abnormalities and AL growth: BVF may reflect an underlying inefficiency or instability in the central visual processing network. This suboptimal neural state may impair the precise cortical control of the accommodation-convergence system, likely leading to an increase in accommodative lag—an established risk factor for myopia progression (18). Persistent accommodative lag, in turn, generates chronic hyperopic defocus signals on the retina. In experimental models, such aberrant signals have been shown to be powerful drivers of scleral remodeling and axial elongation.

The clinical implications of our findings are substantial. First, BVF assessment—particularly using the objective and efficient detection methods employed in this study—may serve as a valuable tool for risk stratification. Routine BVF screening in school-aged children may facilitate the early identification of individuals at high risk for rapid axial growth, thereby enabling timely intervention and targeted management of myopia. Second, our results further support the effectiveness of binocular visual training in delaying axial elongation. Integrating BVF management into existing myopia prevention strategies (e.g., increased outdoor activities, low-concentration atropine) could enhance current approaches and create a more comprehensive, multidimensional strategy for myopia control.

This study has several limitations. First, SE was measured using non-cycloplegic autorefraction, which may explain the lack of a significant relationship between BVF impairment and SE progression. Furthermore, the model did not account for all factors known to influence myopia progression, including genetic predisposition, outdoor activity levels, and interventions such as the use of myopia control lenses and low-concentration atropine. Additionally, the sample was drawn from a single primary school in Shanghai, which may limit the generalizability of the findings.

Future research could employ advanced neuroimaging and ocular biometric techniques, such as cycloplegic autorefraction, to better understand the neurophysiological mechanisms linking BVF impairment to axial elongation. In addition, extending the study duration and incorporating genetic factors, along with a broader range of myopia control measures (such as the use of atropine or myopia control lenses), as well as outdoor activity levels, would facilitate the establishment of long-term associations and provide deeper insights into the impact of BVF impairment on AL, SE, and the AL/CR ratio. Furthermore, future studies could investigate whether BVF abnormalities in childhood serve as predictors for the development of high myopia in adulthood, which would enhance our understanding of the long-term implications of early BVF changes.

## Conclusion

This large-scale longitudinal study demonstrates that BVF impairment, as assessed by the naked-eye 3D visual function examination system, is an independent and significant risk factor for AL elongation in children aged 8–10 years, exhibiting a clear dose-response relationship. This effect is parameter-specific, primarily driving the biological growth of the eye as measured by AL, without showing a significant direct impact on SE or AL/CR ratio progression within this age group. These findings validate BVF screening using this specific methodology as a novel and crucial tool for identifying children at high risk of rapid myopia progression. Consequently, interventions targeting BVF management hold substantial promise as a strategic component of precision myopia prevention and control in this specific pediatric population.

## Data Availability

The raw data supporting the conclusions of this article will be made available by the authors, without undue reservation.

## Supplementary material

Supplementary Figure S1 Longitudinal changes in SE by BVF groups. **(A)** Scatter points and box plots showing the distribution of SE at each time point. The boxes represent the interquartile range, the horizontal line inside the box indicates the median, and the whiskers extend to the minimum and maximum values. **(B)** Line graph showing the mean spherical equivalent with standard error at each time point.

Supplementary Figure S2 Longitudinal changes in AL/CR ratio by BVF groups. **(A)** Scatter points and box plots showing the distribution of AL/CR ratio at each time point. The boxes represent the interquartile range, the horizontal line inside the box indicates the median, and the whiskers extend to the minimum and maximum values. **(B)** Line graph showing the mean AL/CR ratio with standard error at each time point.

Supplementary Figure S3 Forest plot of factors associated with SE change rate. Forest plot showing regression coefficients and 95% confidence intervals for interaction terms in the fully adjusted linear mixed model. Negative values indicate faster myopia progression. The reference groups are: BVF normal, emmetropia, male, lower grades, no strabismus, no astigmatism, and no anisometropia.

Supplementary Figure S4 Forest plot of factors associated with AL/CR ratio change rate. Forest plot presenting regression coefficients and 95% confidence intervals for interaction terms in the fully adjusted linear mixed model. Positive values indicate faster increase in AL/CR ratio. The reference groups are: BVF normal, emmetropia, male, lower grades, no strabismus, no astigmatism, and no anisometropia.

Supplementary Table S1 Linear mixed model results for BVF effects on SE.

Supplementary Table S2 Linear mixed model results for BVF effects on AL/CR ratio.

Supplementary Table S3 Pairwise comparisons of SE change rates among different BVF groups.

Supplementary Table S4 Pairwise comparisons of AL/CR ratio change rates among different BVF groups.

Supplementary Table S5 Clinical significance of SE change rates by different BVF status.

Supplementary Table S6 Clinical significance of AL/CR ratio change rates by different BVF status.
