# Supplementary figures and images for: Binocular visual function impairment is an independent risk factor in axial length growth and myopia progression
Source: Front Med (Lausanne). 2026 Jan 5;12:1738844. doi: 10.3389/fmed.2025.1738844 (PMC12813159; doi:10.3389/fmed.2025.1738844)

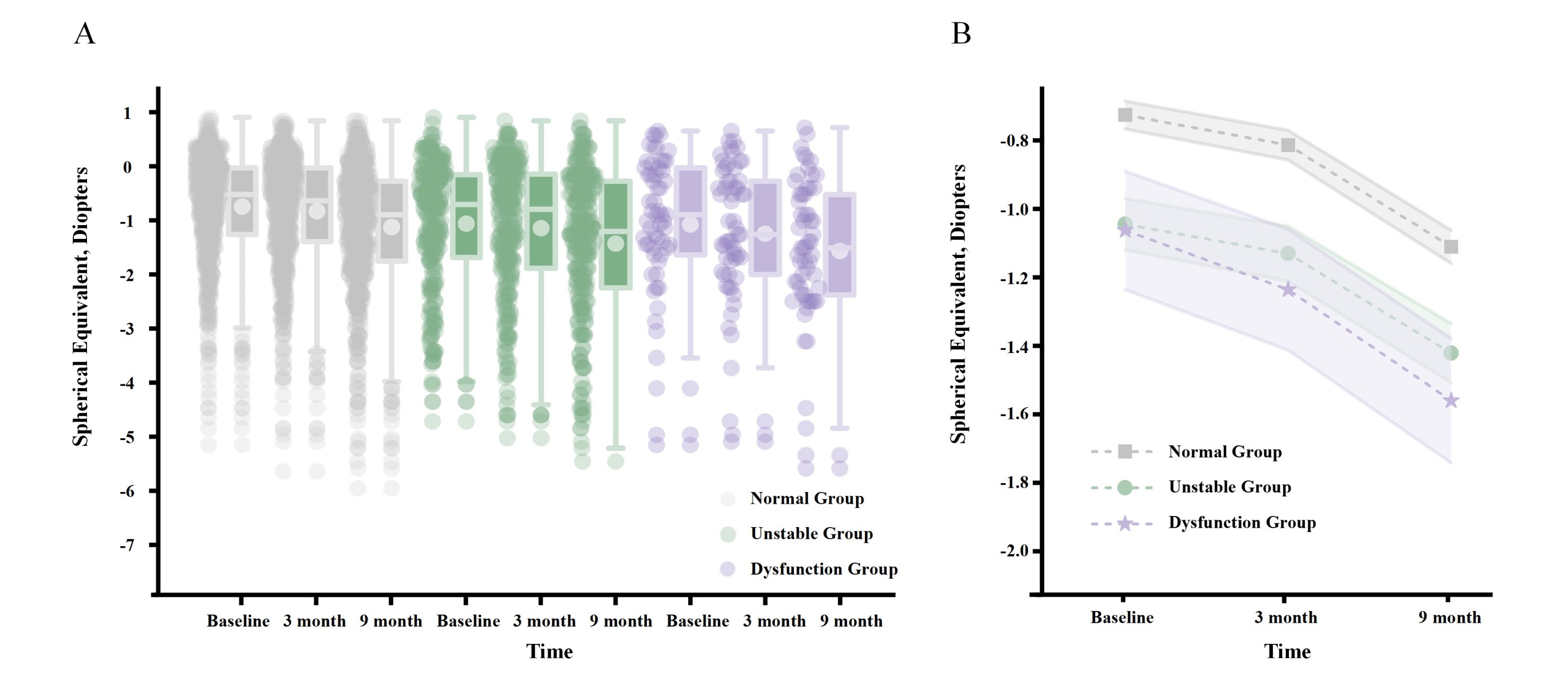

Supplement: Supplementary Figure S1 — Longitudinal changes in SE by BVF groups. (A) Scatter points and box plots showing the distribution of SE at each time point. The boxes represent the interquartile range, the horizontal line inside the box indicates the median, and the whiskers extend to the minimum and maximum values. (B) Line graph showing the mean spherical equivalent with standard error at each time point. [file Image_1.jpeg]

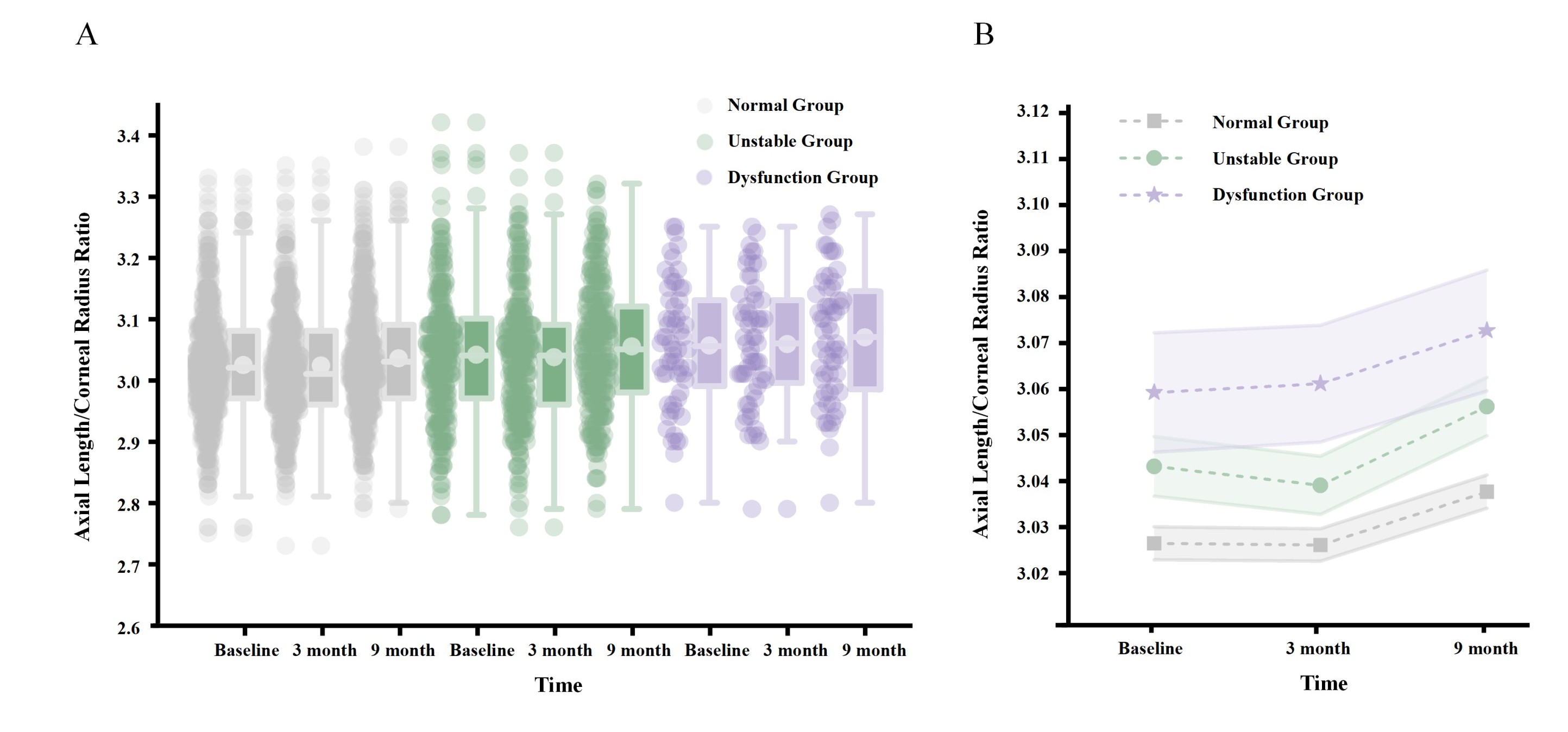

Supplement: Supplementary Figure S2 — Longitudinal changes in AL/CR ratio by BVF groups. (A) Scatter points and box plots showing the distribution of AL/CR ratio at each time point. The boxes represent the interquartile range, the horizontal line inside the box indicates the median, and the whiskers extend to the minimum and maximum values. (B) Line graph showing the mean AL/CR ratio with standard error at each time point. [file Image_2.jpeg]

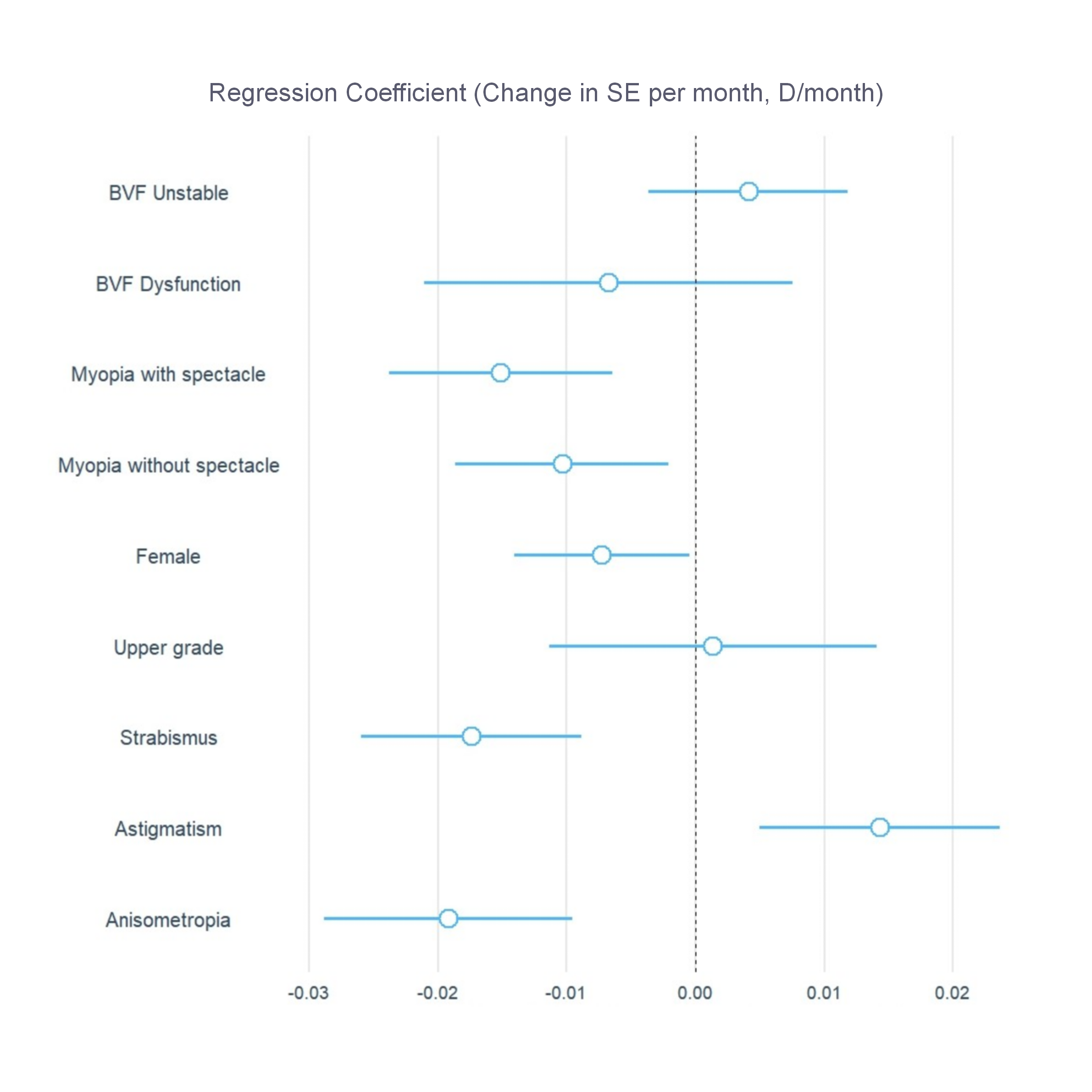

Supplement: Supplementary Figure S3 — Forest plot of factors associated with SE change rate. Forest plot showing regression coefficients and 95% confidence intervals for interaction terms in the fully adjusted linear mixed model. Negative values indicate faster myopia progression. The reference groups are: BVF normal, emmetropia, male, lower grades, no strabismus, no astigmatism, and no anisometropia. [file Image_3.tif]

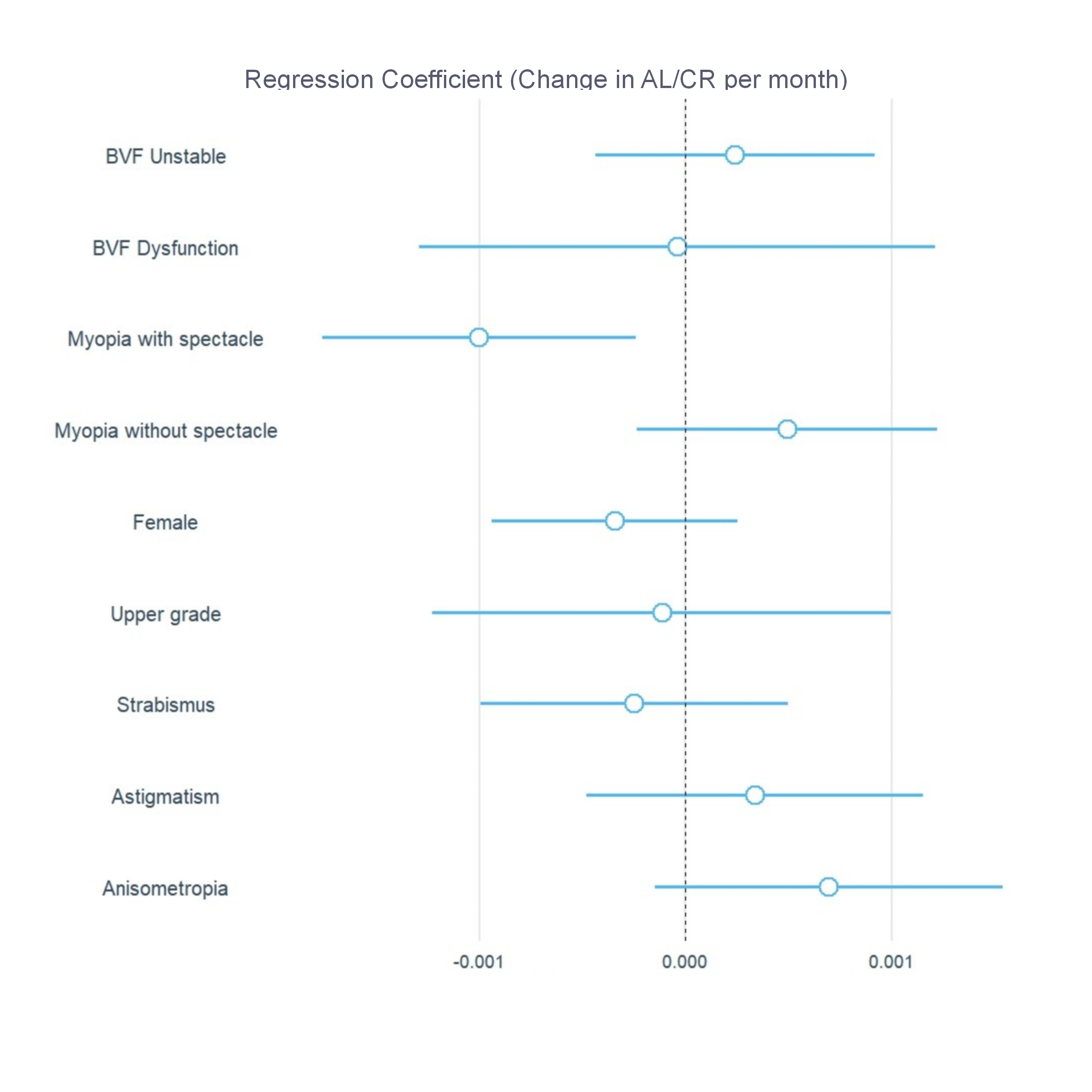

Supplement: Supplementary Figure S4 — Forest plot of factors associated with AL/CR ratio change rate. Forest plot presenting regression coefficients and 95% confidence intervals for interaction terms in the fully adjusted linear mixed model. Positive values indicate faster increase in AL/CR ratio. The reference groups are: BVF normal, emmetropia, male, lower grades, no strabismus, no astigmatism, and no anisometropia. [file Image_4.tif]
