# Supplementary material for: Binocular visual function impairment is an independent risk factor in axial length growth and myopia progression
Source: Front Med (Lausanne). 2026 Jan 5;12:1738844. doi: 10.3389/fmed.2025.1738844 (PMC12813159; doi:10.3389/fmed.2025.1738844)
Supplement: Supplementary Table S1 — Linear mixed model results for BVF effects on SE. [file Table_1.docx]

| **Supplementary Table 1. Linear Mixed Model Results for BVF Effects on SE** | | | |
| --- | --- | --- | --- |
| Variable | Model 1  Coefficients (spherical error) | Model 2  Coefficients (spherical error) | Model 3  Coefficients (spherical error) |
| BVF Unstable (vs Normal) | -0.3186 (0.0855)*** | -0.3277 (0.0848)*** | -0.2113 (0.0648)** |
| BVF Dysfunction (vs Normal) | -0.3565 (0.1582)* | -0.3530 (0.1566)* | -0.2613 (0.1199)* |
| Time trend (per month) | -0.0436 (0.0021)*** | -0.0474 (0.0708) | -0.0445 (0.0702) |
| BVF Unstable × Time | 0.0010 (0.0039) | 0.0015 (0.0040) | 0.0041 (0.0039) |
| BVF Dysfunction × Time | -0.0113 (0.0073) | -0.0115 (0.0073) | -0.0068 (0.0073) |
| AIC | 5380.2 | 5367.1 | 4784 |
| BIC | 5427.8 | 5450.4 | 4926.9 |
| Log Likelihood | -2682.1 | -2669.5 | -2368 |
| Model 1: Basic model Model 2: Adjusted for age, gender, grade; Model 3: Fully adjusted for age, gender, grade, spectacle use, astigmatism, anisometropia, and strabismus *p < 0.05, **p < 0.01, ***p < 0.001  Abbreviation: BVF, binocular visual function; SE, spherical equivalent; AIC, akaike information criterion; BIC, bayesian information criterion. | | | |

| **Supplementary Table 2. Linear Mixed Model Results for BVF Effects on AL/CR Ratio** | | | |
| --- | --- | --- | --- |
| Variable | Model 1  Coefficients (spherical error) | Model 2  Coefficients (spherical error) | Model 3  Coefficients (spherical error) |
| BVF Unstable (vs Normal) | 0.01502 (0.00689)* | 0.01728 (0.00675)* | 0.00910 (0.00572) |
| BVF Dysfunction (vs Normal) | 0.03379 (0.01275)** | 0.03301 (0.01246)** | 0.02699 (0.01060)* |
| Time trend (per month) | 0.00136 (0.00019)*** | 0.00570 (0.00615) | 0.00419 (0.00615) |
| BVF Unstable × Time | 0.00030 (0.00034) | 0.00032 (0.00034) | 0.00024 (0.00035) |
| BVF Dysfunction × Time | 0.00021 (0.00063) | 0.00021 (0.00063) | -0.00004 (0.00064) |
| AIC | -8685.6 | -8720 | -9063.9 |
| BIC | -8638 | -8636.6 | -8921 |
| Log Likelihood | 4350.8 | 4374 | 4555.9 |
| Model 1: Basic model Model 2: Adjusted for age, gender, grade; Model 3: Fully adjusted for age, gender, grade, spectacle use, astigmatism, anisometropia, and strabismus *p < 0.05, **p < 0.01, ***p < 0.001  Abbreviation: BVF, binocular visual function; AL/CR ratio, axial length to-corneal radius ratio; AIC, akaike information criterion; BIC, bayesian information criterion. | | | |

| **Supplementary Table 3. Pairwise Comparisons of SE Change Rates Among Different BVF Groups** | | | |
| --- | --- | --- | --- |
| Group Comparison | Model 1  Coefficients (spherical error) | Model 2  Coefficients (spherical error) | Model 3  Coefficients (spherical error) |
| Normal vs Unstable | -0.00096 (0.00395) | -0.00150 (0.00395) | -0.00410 (0.00394) |
| Normal vs Dysfunction | 0.01131 (0.00731) | 0.01147 (0.00730) | 0.00679 (0.00730) |
| Unstable vs Dysfunction | 0.01227 (0.00773) | 0.01297 (0.00773) | 0.01088 (0.00766) |
| all comparisons FDR-adjusted. *p < 0.05, **p < 0.01, ***p < 0.001  Abbreviation: BVF, binocular visual function; SE, spherical equivalent. | | | |

| **Supplementary Table 4. Pairwise Comparisons of AL/CR Ratio Change Rates Among Different BVF Groups** | | | |
| --- | --- | --- | --- |
| Group Comparison | Model 1  Coefficients (spherical error) | Model 2  Coefficients (spherical error) | Model 3  Coefficients (spherical error) |
| Normal vs Unstable | -0.000303 (0.000342) | -0.000322 (0.000343) | -0.000241 (0.000345) |
| Normal vs Dysfunction | -0.000215 (0.000634) | -0.000211 (0.000634) | 0.000040 (0.000639) |
| Unstable vs Dysfunction | 0.000088 (0.000671) | 0.000111 (0.000671) | 0.000281 (0.000671) |
| all comparisons FDR-adjusted. *p < 0.05, **p < 0.01, ***p < 0.001  Abbreviation: BVF, binocular visual function; AL/CR ratio, axial length to-corneal radius ratio. | | | |

| **Supplementary Table 5. Clinical Significance of SE Change Rates by Different BVF Status** | | | |
| --- | --- | --- | --- |
| BVF Group | Monthly Growth Rate(mm/month) | Annual Growth Rate(mm/year) | Fold Change vs Normal |
| Normal | -0.0545 D/month | -0.6536 D/year | 1 |
| Unstable | -0.0504 D/month | -0.6045 D/year | 0.92 |
| Dysfunction | -0.0613 D/month | -0.7351 D/year | 1.12 |
| Based on marginal means from fully adjusted model (Model 3)  Abbreviation: BVF, binocular visual function; SE, spherical equivalent. | | | |

| **Supplementary Table 6. Clinical Significance of AL/CR ratio Change Rates by Different BVF Status** | | | |
| --- | --- | --- | --- |
| BVF Group | Monthly Growth Rate(mm/month) | Annual Growth Rate(mm/year) | Fold Change vs Normal |
| Normal | 0.00161/month | 0.0194/year | 1 |
| Unstable | 0.00185/month | 0.0223/year | 1.15 |
| Dysfunction | 0.00157/month | 0.0189/year | 0.98 |
| Based on marginal means from fully adjusted model (Model 3)  Abbreviation: BVF, binocular visual function; AL/CR ratio, axial length to-corneal radius ratio. | | | |
